# A method to reduce imbalance for site-level randomized stepped wedge implementation trial designs

**DOI:** 10.1186/s13012-019-0893-3

**Published:** 2019-05-03

**Authors:** Robert A. Lew, Christopher J. Miller, Bo Kim, Hongsheng Wu, Kelly Stolzmann, Mark S. Bauer

**Affiliations:** 1 VA Boston Healthcare System, Center for Healthcare Organization and Implementation Research, 150 South Huntington Avenue, Jamaica Plain, Boston, MA 02130 USA; 2 The Massachusetts Veterans Epidemiology Research and Information Center, 150 South Huntington Avenue, Jamaica Plain, Boston, MA 02130 USA; 3 Department of Psychiatry, Harvard Medical School, Boston, MA USA; 4 Department of Computer Science & Networking, Wentworth Institute of Technology, Boston, USA

**Keywords:** Stepped wedge, Imbalance, Nested control trails

## Abstract

**Background:**

Controlled implementation trials often randomize the intervention at the site level, enrolling relatively few sites (e.g., 6–20) compared to trials that randomize by subject. Trials with few sites carry a substantial risk of an imbalance between intervened (cases) and non-intervened (control) sites in important site characteristics, thereby threatening the internal validity of the primary comparison. A stepped wedge design (SWD) staggers the intervention at sites over a sequence of times or time waves until all sites eventually receive the intervention. We propose a new randomization method, sequential balance, to control time trend in site allocation by minimizing sequential imbalance across multiple characteristics. We illustrate the new method by applying it to a SWD implementation trial.

**Methods:**

The trial investigated the impact of blended internal-external facilitation on the establishment of evidence-based teams in general mental health clinics in nine US Department of Veterans Affairs medical centers. Prior to randomization to start time, an expert panel of implementation researchers and health system program leaders identified by consensus a series of eight facility-level characteristics judged relevant to the success of implementation. We characterized each of the nine sites according to these consensus features. Using a weighted sum of these characteristics, we calculated imbalance scores for each of 1680 possible site assignments to identify the most sequentially balanced assignment schemes.

**Results:**

From 1680 possible site assignments, we identified 34 assignments with minimal imbalance scores, and then randomly selected one assignment by which to randomize start time. Initially, the mean imbalance score was 3.10, but restricted to the 34 assignments, it declined to 0.99.

**Conclusions:**

Sequential balancing of site characteristics across groups of sites in the time waves of a SWD strengthens the internal validity of study conclusions by minimizing potential confounding.

**Trial registration:**

Registered at ClinicalTrials.gov as clinical trials # NCT02543840; entered 9/4/2015.

## Background

Balancing comparison groups is a longstanding challenge for both controlled trials and observational studies. The issue is particularly acute in designs that randomize by site. Typically, subject-level randomization designs draw from a large pool, while site-level randomization draws from a much smaller pool, thereby increasing the risk of imbalance.

Stratified randomization is impractical when the site profile contains multiple relevant healthcare site characteristics, each of which would ideally be balanced across study conditions. Similarly, matching site-for-site often entails questionable assumptions, namely that all characteristics have equal weight, are mutually independent, and are exchangeable. For example, two sites in the same region may serve socio-economically diverse populations, but two from different regions may serve socio-economically similar populations. In that case, the research team would need to decide whether stratifying based on region or socio-economic status would better serve the study goals.

Sequential balance is a form of balance quite different from mean balance, which seeks to balance characteristics across distinct groups (e.g., control and intervention sites). The need for sequential balance arises within cluster designs such as a stepped wedge design (SWD) where intervention cannot be done at all sites at one time, but instead, the intervention is staggered over a sequence of times or time waves. For example, suppose a SWD with three time waves hopes to reduce the hospitalization rate. If the study sites in wave 1 lie in Region 1, in wave 2 lie in Region 2, and in wave 3 lie in Region 3, then the two predictors, “time” and “Region,” are confounded. An optimal remedy places sites from all three Regions in every wave. We use the term “confounding” to connote only “ambiguity in interpretation” but not a variable that distorts the association between a causal factor and an outcome. Time is calendar time, the dates at which the intervention begins in each of the time waves. If the three waves start at 4-month intervals, then subtracting the time when wave 1 starts from each of the three start times, the values of time shift to become 0, 4, and 8 months. The concept of “time trend” underlies the sequential imbalance score. Time trend indicates the size of the correlation between calendar time and a continuous site characteristic such as the number of available hospital beds. Correlation is invariant under shifts in a variable, so we can regard wave 1 as starting at time 0. However, while a variable with three or more categories may be confounded with time, the correlation between it and the variable “time” is not well defined. Thus, we extend the concept of time trend to a categorical variable in the “Methods” section and thereby define the imbalance score for categorical characteristics.

Sequential balance among site characteristics reduces confounding and hence potential bias as is the aim in a quasi-experimental design [1]. It complements mean balance for parallel group designs, in which some sites receive and other sites do not receive the intervention. Perfect mean balance over time waves not only implies perfect sequential balance, but also perfect balance for nearly any other time pattern. The added covariate “time” might produce a linear pattern of time trend with a site characteristic. Then one might wish to simultaneously balance both means and sequences to include mean balance to protect against other time patterns. We address this tradeoff between types of balance after describing sequential balance.

Sequential balance draws on a method developed nearly 80 years ago by RA Fisher, who proposed balanced incomplete block (BIB) designs [2] to refine analysis of variance [3]. Indeed, a SWD can be cast as a BIB design with waves as blocks and interventions as treatments [4, 5]. A BIB design restricts the set of patterns of treatment assignments to blocks. Sequential balance restricts the set of patterns of site assignment to a subset of site assignments with the lowest imbalance scores.

The rest of the paper elaborates on this method. The “Methods” section describes the study in which we utilized this balance method, formally defines sequential balance, and offers a candidate for the imbalance score. The “Results” section applies our method to the study and presents the results it yielded. The “Discussion” section elaborates on extensions and describes limitations of the method.

## Methods

### Study, sites, and characteristics

The example herein comes from the recently concluded Behavioral Health Interdisciplinary Program-Collaborative Care Model (BHIP-CCM) implementation trial, a study of nine general mental health clinics within US Department of Veterans Affairs (VA) hospital facilities [6], a mixed research/quality improvement project approved by the VA Central Institutional Review Board. The implementation strategy, blended internal-external facilitation [7], was designed to implement the evidence-based collaborative care model for mental health conditions [6, 8, 9] and thus to improve staff performance and patient health status as measured by changes over time. This SWD staggered the start of implementation over three time waves, thereby randomizing the implementation start times, with three sites beginning at each of three start times. Thus, over the course of the study, every site received the implementation intervention.

Sites were recruited via national search, by contacting national VA mental health leadership, then soliciting interest from each VA region, then engaging sites on an individual basis. Study investigators had not worked with any of the sites previously. Through this process, 12 sites agreed to participate and the first nine to complete the application were enrolled. One site dropped out prior to randomizing and was replaced by one of the three remaining (*n* = 9 total).

Site characteristics for balancing were identified by a consensus of national VA mental health leadership involved in guiding the BHIP-CCM program, implementation researchers, and clinical trialists. In a half-day meeting, participants brainstormed facility characteristics they judged likely to be relevant to the success of BHIP-CCM implementation. Twenty characteristics were identified. Implementation researchers then identified relevant measures, where available; eliminated highly collinear measures; and reviewed the resultant list with BHIP-CCM leadership. The final list comprised eight site characteristics, summarized in Table 1. Each of the nine sites was characterized according to these features. The resultant sample profile, and comparison to VA facilities nationally, is found in Table 2.

**Table 1:** Eight site factors to be balanced over time in the BHIP-CCM study

Continuous factors	Categorical/ordinal factors
All-Employee Survey item on psychological safety	VA geographic region
Number of BHIP teams previously established	Urban/Rural/Highly Rural
Number of patients seen in general mental health clinics in the prior year	VA facility complexity category (a five-level index including relevant characteristics such as array of available services, inpatient care intensity, size of training programs, etc.)
Percentage of primary care patients seen in integrated primary care/mental health teams (an index of system redesign experience)	–
Proportion of clinic visits conducted via telephone (an index of use of non-traditional treatment methods)	–

**Table 2:** Profile of characteristics for the nine study sites and 118 national sites

Site characteristic	Median and range of sample (*n* = 9)	Median and range of VA facilities nationally (*n* = 118)
All-Employee Survey item on psychological safety	3.67 (3.55–3.83)	3.67 (3.36–3.97)
Number of BHIP teams previously established	4 (1–7)	3 (0–16)
Number of patients seen in general mental health clinics in the prior year	8351 (2239–26,115)	7976 (1477–29,166)
Percentage of primary care patients seen in integrated primary care/mental health teams	7.78% (2.03%–12.79%)	7.24% (1.82%–13.20%)
Percentage of clinic visits conducted via telephone	18% (9%–29%)	16% (3%–87%)
VA geographic region, *n* (%)		
Region 1	0 (0%)	28 (23.73%)
Region 2	3 (33.33%)	33 (27.97%)
Region 3	3 (33.33%)	35 (29.66%)
Region 4	3 (33.33%)	22 (18.64%)
Rural, *n* (%)	1 (11.11%)	20 (16.95%)
VA facility complexity, *n* (%)		
Complexity 1a (most complex)	1 (11.11%)	34 (28.81%)
Complexity 1b	2 (22.22%)	18 (15.25%)
Complexity 1c	3 (33.33%)	20 (16.95%)
Complexity 2	1 (11.11%)	21 (17.80%)
Complexity 3 (least complex)	2 (22.22%)	25 (21.19%)

### Sequential balance differs from other types of balance

Sequential balancing reduces time trend of site characteristics over the times of the SWD time waves. For a characteristic such as facility number of available beds, three time-wave groups mean balance if the mean numbers of beds across the facilities are equal, e.g., “300, 300, 300.” In the special case that each wave contains the same number of sites, sequential balance coincides with removing the pattern of time trend from the sequence of time wave means and another site characteristic. For example, mean number of beds rises if the time sequence of means is “100, 200, 300.” In contrast, an oscillating time sequence of means, such as “300, 100, 300,” is not mean balanced, but has no strong pattern of time trend with number of beds and is sequentially balanced, so that mean number of beds is not strongly correlated with time. The term “balance” in “sequential balance” is used here because the sequence can be visualized as weights balanced on a seesaw at varying distances from the fulcrum; that is, for the sequence “300, 100, 300”, the seesaw is balanced with a weight of 100 at the fulcrum and weights of 300 one unit away on each side of the fulcrum.

To sequentially balance as many site characteristics as possible, we want to have many ways as possible to assign the sites to waves.

#### Example 1

Consider a SWD with six sites that assigns two sites to each of three waves. There are 90 = 6!/(2! 2! 2!) distinct site assignment patterns. If the six sites have a number of beds of 100, 300, 300, 300, 300, and 300, then no site assignment can be mean balanced because of the outlier value 100. However, the seesaw analogy shows that after placing the site with value 100 in the middle wave (at the fulcrum), then 30 of the 90 site assignments achieve sequential balance. Also, all 90 site assignments have the same mean balance, so that adding sequential balance does not alter the mean balance. However, exact mean balance is too stringent a criterion for sequential balance.

With so few sites and so many characteristics to balance, it was essential to simultaneously minimize sequential imbalance to reduce the time trend for as many site characteristics as possible across the three start-time groups. Because of these basic differences, a review of the rich literature on methods to simultaneously mean balance many characteristics showed that they did little to improve sequential balance. Rosenbaum, Ross, and Silber proposed the *fine balance* method for medical outcome analyses that profile and rank hospital performance [10]. This method could simultaneously balance means for up to 60 factors by having a computer algorithm systematically search a large pool of potential controls. These authors have developed many approaches to mean balance and matching [11–13]. Zubizaretta [14] proposed *stable balancing weights*, a method that reweights the control group to create mean balance, but reweighting does not help to reduce sequential imbalance. Both methods were developed to complement the propensity score methods put forth by Rosenbaum and Rubin [15]. Also, other methods [16–18] have been used to mean balance case and control sites in cluster randomized trials, but a SWD need not have any “control” clusters.

A large pool of potential sites that provides near exact mean balance removes both outliers (as in Example 1) and the need for sequential balance. Exact mean balance allows one to permute the sites among the waves without changing mean balance. In other words, exact mean balance implies exact sequential balance. However, a small pool of sites makes it likely that some characteristic means will be outliers. In this case, a sound strategy begins with seeking mean balance and then adjusting the assignment of sites to waves to improve sequential balance while only slightly perturbing mean balance as in Example 1.

Propensity score methods are not applicable to sequential balance. In a comparative study, the feature, disease status, distinguishes cases from controls. Typically, a propensity score is the predicted probability based on the site characteristics of the chance (or propensity) of receiving treatment for the disease. An untreated control with the same propensity score then resembles a treated case. Matching on the score thereby avoids the hard task of matching on each of the multiple characteristics summarized in the score. The time waves of a SWD have no distinguishing feature such as “disease treatment” on which to base a propensity score. In other words, the a priori propensity of assigning a site to either the first or to the second wave is always one half because the wave number does not depend on any of the site characteristics.

### The imbalance formula

An imbalance score (IMB) is a weighted sum of terms, Imb, one term for each characteristic of interest. All continuous characteristics were rescaled to have standard deviation one to make them more homogeneous. The general theory below considers both continuous and categorical characteristics. However, the BHIP-CCM study used tertiles to transform all continuous factors into three-category factors because continuous characteristics intrinsically have more weight than categorical ones. Simulations for a continuous form and the three-category form showed that on average the imbalance scores for the continuous form were 50% larger. Recently, we have developed a conjecture that for a *K*-category form, the continuous form has an average imbalance score larger by the ratio *K*/(*K* − 1).

First, we develop a formula for imbalance, Imb(*Y*), for a continuous characteristic, *Y*.

A typical SWD has *W* > 1 waves at regular time intervals, so that time *T* = 1, 2, … , *W*. A total of *n* sites are assigned to (or distributed over) the *W* waves. Time trend is defined by the slope, *β*, of the regression line, *Y* = *α* + *βT*. A flat line with *β* = 0 shows no time trend. Observing variable, *Y*, at *n* sites, the slope estimate, $\widehat{\beta}=0$ if ${\sum}_{i=1}^n{Y}_i{t}_i=0$, where ${t}_i={T}_i-\overline{T}$ is the deviation from the mean, $\overline{T}$. In terms of the seesaw analogy, *Y*_*i*_ is the weight on the seesaw and *t*_*i*_ is the distance from the fulcrum. We define:

1 $$ \mathrm{Imbalance}\ \mathrm{for}\ \mathrm{continuous}\ \mathrm{characteristic},\kern0.5em Y,\mathrm{is}\kern0.5em \mathrm{Imb}(Y)=\mid {\sum}_{i=1}^n{Y}_i{t}_i\mid . $$

Second, we develop a formula for Imb(*Y*) for a categorical characteristic, *Y*. Consider the characteristic, Urban/Suburban/Rural (USR). Regarding each category as a binary variable, then let *U* = 1 if the site is urban and *U* = 0 otherwise, *S* = 1 if suburban and *S* = 0 otherwise, and *R* = 1 if rural and *R* = 0 otherwise. As in Eq. (1), the imbalance for the category “Urban” is $\mathrm{Imb}(U)=\mid {\sum}_{\mathrm{i}=1}^{\mathrm{n}}{U}_i{t}_i\mid$ and, by extension, the imbalance for the characteristic, USR, is

$$ \mathrm{Imb}\left(\mathrm{USR}\right)={f}_U\left|{\sum}_{i=1}^n{U}_i{t}_i\right|+{f}_S\left|{\sum}_{i=1}^n{S}_i{t}_i\right|+{f}_R\left|{\sum}_{i=1}^n{R}_i{t}_i\right|, $$

where *f*_*U*_, *f*_*S*_, and *f*_*R*_ are the *overall* relative frequencies of the categories.

In general, an imbalance score for a *K*-category characteristic, *Y*, with category relative frequencies, *f*_1_, *f*_2,_ … , *f*_*K*_, is the sum,

$$ \mathrm{Imb}(Y)={\sum}_{k=1}^K{f}_k\mid \mathrm{Imb}\mathrm{alance}\ \mathrm{for}\ \mathrm{category}\ k\mid . $$

Finally, after the user subjectively assigns relative weights, *w*, to each characteristic, the overall weighted imbalance score for *j* = 1 to *J* characteristics is

2 $$ \mathrm{IMB}={\sum}_{j=1}^J{w}_j\mathrm{Imb}\left({Y}_j\right). $$

Our imbalance formula for a site characteristic is based on the simple linear regression model, *Y* = *α* + *βT*, that makes no sense if *Y* has three or more categories. Thus, each category is treated as a binary variable, thereby evaluating sequential imbalance as a weighted sum of time trends, each term in the sum based on a linear regression model with respect to a binary variable, *Y*, predicted by time, *T*. The binary variables are linearly dependent. For example, a variable *Y* such as “Gender” can be coded either as {*Y* = 1 if male and *Y* = 0 if female} or, in reverse, coded { *Y* = 1 if female and *Y* = 0 if male}. The two forms have opposite estimated slopes that are either *b* or − *b*. Weighting by the observed proportions of males and females, *p*_*m*_ and *p*_*f*_, the imbalance score is proportional to *p*_*m*_|*b*| + *p*_*f*_|*b*| =  ∣ *b*∣, the same result obtained by removing the linearly dependent category from the evaluation. The imbalance formula is correct for any number of categories, there is no need to remove a category, and the binary time-trend regression equations make sense.

Confounding is not explicitly related to time but arises when two predictors of a study outcome are nearly surrogates for each other. If a study outcome such as the number of hospitalizations, *H*, rises over time, *T*, then a characteristic such as a categorical variable, USR, with site-categories “urban,” “suburban,” and “rural” might also change with time, *T* (e.g., all sites in wave 1 are “rural,” all in wave 2 are “suburban,” and all in wave 3 are “urban”). The total variation in the model equals variation explained by the model plus unexplained variation, where the ratio of explained over total is the proportion of total variation (*R*^2^). In an efficient model, each added variable markedly increases *R*^2^. However, when the values of *R*^2^ for the one-factor models “*H* = *T*” and “*H* = USR” are about equal and the value of *R*^2^ for the two-factor model “*H* = *T* + USR” is only a bit larger, then the second factor adds little, implying it is a redundant confounder, because we cannot tell if hospitalizations actually rose over time or if this is merely an artifact of USR.

The search to minimize IMB used simple random sampling without replacement (SRS) to generate sequences of 9 digits. Sites numbered 1 to 9 were assigned to 3 waves each with 3 sites. The sequence (1 2 3) (4 5 6) (7 8 9) indicates that the first wave is sites 1, 2, 3, the second is sites 4, 5, 6, and the third is sites 7, 8, 9. Redundant sequences, e.g., (3 2 1) (6 5 4) (9 8 7), produce the same 3 waves. A simple program can remove such redundancy. For the BHIP-CCM trial, only *N* = 1680 distinct sequences covered all possible distinct assignments to three waves, but with a pool of 100 sites, *N* > 10^14^. However, with only 10,000 SRS sequences, one can find an assignment with a near-minimum imbalance score. For example, suppose a near minimum rarely occurs with probability, 0.0005, then the probability of finding at least one among 10,000 SRS is 99%.

The search for the minimum imbalance score had three steps; (1) generate a large list of sequences using SRS, (2) evaluate the imbalance score for each sequence, and (3) randomly select one sequence from those with the lowest scores. More sophisticated or “smart” search methods, such as the Newton-Raphson method [19], accelerate the search by “learning” from each evaluation where best to look next for the minimum. However, a smart search can fail if it is ill-suited to the formula for the imbalance score. The slower but sure SRS can nearly minimize any formula for the imbalance score, unless “near minimum” is infinitesimally rare.

In general, one should prefer mean balance over time waves because nearly exact mean balance implies nearly exact sequential balance. Preference can be expressed as an explicit loss function, *L*, in the form:

$$ L\left(\omega \right)=\mathrm{Loss}\left(\mathrm{imperfect}\ \mathrm{mean}\ \mathrm{balance}\right)+{\omega}^{\ast}\kern0.5em \mathrm{Loss}\left(\mathrm{imperfect}\ \mathrm{sequential}\ \mathrm{balance}\right). $$

The mean-balance term might be the sum over all characteristics of the squared deviations of the time wave means from the grand mean over all the time waves. Then, for *n* characteristics, { *Y*_1_, *Y*_2_, … , *Y*_*n*_} and time waves (*w* = 1, 2, …*W*);

$$ \mathrm{Loss}\left(\mathrm{imperfect}\ \mathrm{mean}\ \mathrm{balance}\right)={\sum}_{i=1}^n{\sum}_{w=1}^W{\left(\ {\overline{Y}}_{i,w}-{\overline{Y}}_i\right)}^2\ \mathrm{where}\ {\overline{Y}}_i\ \mathrm{is}\ \mathrm{the}\ \mathrm{grand}\ \mathrm{mean}\ \mathrm{of}\ {Y}_i. $$

The sequential balance term might be the imbalance score, IMB given by Eqs. 1 and 2. Given loss functions calibrated to have similar scales, a user-chosen value of the weight *ω* = 0.2 in *L*(*ω*) would favor mean balance, whereas *ω* = 5 would favor sequential balance.

The tradeoff between mean balance and sequential balance has a weight or “tuning parameter” omega (*ω*), but how to choose omega is an open question [19]. Our trade-off formula takes the same form if one views mean imbalance as the primary loss function and views sequential imbalance as a penalty function. Implicitly, mean balance is more important, while varying omega indicates how well the various trade-offs work. We propose first finding a subset of site assignments that have low mean-imbalance scores and then searching over the subset for site assignments that have low imbalance scores. Example 1 shows that if mean imbalance varies little within the subset then values such as *ω* = 5 would more easily distinguish site assignments with low sequential imbalance.

Cast as *constrained optimization* [19], the weight *ω* = 0.2 would minimize mean-balance subject to a constraint on sequential balance and the weight *ω* = 5 would minimize sequential balance subject to a constraint on mean balance. In either case, for each choice of *ω*, we wish to assign sites to time waves that minimize the loss, *L*(*ω*). Robust designs nearly minimize *L*(*ω*) for many choices of *ω*. In the number of beds example in the “Background” section, an outlier (number of beds = 100) hindered mean balance, yet many designs had perfect sequential balance regardless of the choice of *ω*.

Linear time trend is only one possible time pattern. Only mean balance protects against all patterns. Sequential balance might worsen matters if the outcome response first rose and then fell in a three-wave study. When mean balance is poor and linear time trend very likely, then one should choose a value such as *ω* = 5, but with near-perfect mean balance, one should choose a value such as *ω* = 0.2.

The loss function, *L*(*ω*), can expand to address several more concerns (that is, additional constraints), but then for each concern one needs to choose a weight and formula for loss. Also, the formulas should have a reasonably common scale.

## Results

In calculating the imbalance score for our SWD trial, the primary set of factor representations used equal weights and re-expressed all continuous characteristics as categorical variables using the tertiles of the continuous variables to define the categories, as summarized above. The overall imbalance score, IMB, was computed using Eq. (1). Coarsening continuous factors into three categories defined by tertiles provided some assurance that the eight factors in the overall score had reasonably similar weights within the formula in Eq. (2).

Table 3 shows the effect of restricting the random selection of the site assignment to the 34 site assignments with the smallest imbalance scores, compared to IMB for all 1680 site assignment schemas. This arbitrary choice of 34 site assignments came at a natural break point in the ordered list of scores and corresponded to approximately the 2% least imbalanced assignments (a pre-determined 10% cutoff might be better). For each of the eight site characteristics in Table 1, restricting possible assignments of sites greatly reduced imbalance, from 3.10 to 0.99 in arbitrary IMB units. From this final group of 34 assignment schemes, one was randomly chosen and utilized to develop the implementation intervention schedule for the trial.

**Table 3:** Imbalance score by site characteristic and overall for all 1680 assignments and for the 34 assignments with the lowest overall imbalance scores

Site characteristics (categorical)	Imbalance score (IMB) for all 1680 site assignments Mean (SD)	Imbalance score (IMB) for 34 site assignment with minimal scores Mean (SD)
Psychological safety	0.40 (0.42)	0.12 (0.11)
Number of BHIP teams previously established	0.38 (0.40)	0.15 (0.13)
Number of patients seen in general mental health clinics in the prior year	0.36 (0.34)	0.09 (0.07)
System redesign experience	0.40 (0.42)	0.10 (0.07)
Proportion of care via telephone	0.38 (0.40)	0.13 (0.11)
VA geographic region	0.40 (0.42)	0.09 (0.07)
Urban/Rural/Highly Rural	0.40 (0.42)	0.18 (0.15)
VA facility complexity category	0.38 (0.40)	0.13 (0.11)
Overall imbalance score	3.10 (0.91)	0.99 (0.32)

An efficient sequential balance over three waves tends to assign sites with outlier values to the middle wave 2 and assign sites without outlier values to waves 1 and 3. In contrast, if a large pool of sites contains nine sites that are nearly exactly mean balanced for all characteristics, then nearly any assignment to the three waves will preserve nearly exact mean balance. Sequential balance helps if the pool of sites is too small to avoid outlier values as illustrated by Example 1, where sequential balanced forced the outlier site into wave 2. Table 4 indicates how in the BHIP-CCM study outliers in site 1 and in site 6 assigned these sites wave 2, whereas sites 8 and 9 that were free of outliers almost always were assigned to waves 1 and 3. The frequencies in Table 4 for waves 1 and 3 are exactly the same because any assignment of sites to waves, if assigned in reverse order, must have the same imbalance score.

**Table 4:** Frequency with which each of the 9 sites in the BHIP-CCM study was assigned to waves 1, 2, and 3 among the 34 site assignments with minimal imbalance scores

Frequency Column Pct	Site 1	Site 2	Site 3	Site 4	Site 5	Site 6	Site 7	Site 8	Site 9
Wave 1	9 26.47	11 32.35	9 26.47	9 26.47	11 32.35	7 20.59	13 38.24	17 50.00	16 47.06
Wave 2	16 47.06	12 35.29	16 47.06	16 47.06	12 35.29	20 58.82	8 23.53	0 0.00	2 5.88
Wave 3	9 26.47	11 32.35	9 26.47	9 26.47	11 32.35	7 20.59	13 38.24	17 50.00	16 47.06
Total	34	34	34	34	34	34	34	34	34

## Discussion

### Summary of method and results

The critical task of minimizing mean imbalance across treatment arms in a controlled trial can be difficult when the number of trial participants is small. This is particularly relevant to implementation controlled trials that randomize a relatively small number of sites to different implementation arms. Similar challenges result when proposing a method to find site assignments that are sequentially well-balanced for multiple site characteristics. To implement our method, the research team created a list of characteristics, agreed on a primary representation, and kept track of the most difficult choices for (1) which to include, (2) which to transform, (3) how to collapse categories, (4) how to deal with ordinal factors, and (5) what factor weights to put in the imbalance score. Then, a search over many SRS site assignments identified a small set of well-balanced site assignments. One set of site assignments was then randomly selected to nearly minimize imbalance, thereby avoiding subjective and forced choices (e.g., stratification) that might introduce bias into the results.

### Sequential balance in the context of other methods to reduce imbalance

Sequential balance of waves in a SWD has advantages over stratification or matching approaches in that these are extremely limited in the number of characteristics they can balance simultaneously. The difficulty arises when multiple relevant characteristics are identified, the pool of sites is small, and stratification on several characteristics would create too many substrata—as is the case in many implementation trials that randomize at the site level. Our method identifies allocation schemas that simultaneously minimize *overall* imbalance among *multiple* characteristics. No single, individual characteristic is necessarily perfectly balanced, which would be at the cost of others (as occurs in simple stratification), but the overall schema or schemas achieve improved multi-way balance over several relevant characteristics. Even if one site characteristic played an identifiable, predominant role, it would make sense to assign it to a middle “fulcrum” time wave rather than assign it to the first or last time wave and “tilt the seesaw.”

As noted, one cannot construct a propensity score for time-wave groups, but the overall imbalance score, IMB, in Eq. (2) serves the same general purpose by forming a composite sum of imbalance scores for each characteristic, Imb(*Y*), as in Eq. (1). Minimizing the sum, IMB, tends to force each term, Imb(*Y*), closer to zero. Moreover, the IMB formula includes a few of the most crucial characteristics rather than the entire set of all available characteristics.

This method for sequential balance of waves in a SWD contrasts with the more stringent traditional method of mean balance. Both mean balance and sequential balance are alike in that each assigns sites to study groups seeking to minimize a sum of terms, one term for each site characteristic. The mean-balance term contains the pairwise differences of the treatment group means for the characteristic. The sequential balance term focuses on the time trend over the time-wave groups for each characteristic. Nevertheless, while the formulas and groups differ, our computational approach to minimization is nearly the same. Because the pool of sites tends to be small, we elected a direct search instead of a faster smart search suited to the formula we wished to minimize. This empowers a researcher to carry out, for example, median balance instead of the mean balance without having to find an algorithm suited to medians. Thus, one can consider enhancing the imbalance formula with constraints such as mean balance across the time waves as in the equation for the loss function, *L*(*ω*); that is, constrained optimization [19] that both mean balance and sequentially balance sites. A SWD cluster randomized study with both intervened (cases) and non-intervened (control) sites would need a constraint to balance the means of cases and controls. Also, one would sequentially balance the site characteristic “case or control” to avoid assigning all case sites to the first wave and all control sites to the last wave. Overall, though, while sequential balancing is based on a different method than mean-balance methods, these same principles can be applied to parallel group randomized controlled designs, and even observational designs.

The methods of Silber et al. [10–13] and our method complement each other; the former precisely matching a large stock of characteristics across a large number of participating subjects and the latter less precisely sequentially balancing eight characteristics across three time waves. Our method is best suited to designs with a small pool of sites while their methods typically draw from a large pool of control subjects and extensive patient record data.

In general, our approach can be applied to balance groups in observational as well as randomized trial designs. For instance, in a secondary analysis within the BHIP-CCM study, the approach was used to identify a control group of 27 VA sites, drawn from 140 potential sites, in order to pair-match 3:1 with the nine chosen study sites. We chose a set of crucial site characteristics and for each characteristic computed the absolute difference between the study site and each potential control site. Finally, we minimized the sum of the absolute differences over all potential control sites.

### Limitations

Several limitations are worth noting. First, sites can only be balanced on characteristics that are measurable and available. There will always be unmeasured—and sometimes unmeasurable—characteristics that cannot be balanced. Analyses of controlled trials can account to some degree for such unmeasured qualities by including sites as fixed or random effects.

Second, the method depends on choosing which characteristics to balance, as well as specifying the weights for the imbalance characteristics. Dependence on the appropriate choice of characteristics also applies to traditional methods such as stratification and matching, and depends on the expertise of the research group. In fact, this sequential balance method accommodates a larger number of characteristics than can stratification or matching, making the tricky choice of one or two stratification or matching variables less risky. Note also that qualitative methods may provide richer profiles of individual sites prior to randomization, and insofar as qualitative themes can be translated into quantitative measures, they may enhance site balancing algorithms. It should also be noted that the site characteristics utilized in this particular study were identified for the purposes of this study and will not necessarily be useful in other studies.

Third, while the use of SRS direct search removes the need for specialized search algorithms to minimize imbalance, some trial and error may arise in deciding how many SRS sequences to draw. Future work can extend the software, but cannot eliminate the need for content expertise and careful decisions about which variables to include that seem most likely to improve balance.

## Conclusions

Implementation trials typically work with relatively small numbers of sites, and the risk of imbalance across intervention arms is correspondingly relatively great. Randomization of small numbers of sites cannot be relied upon to yield homogeneous groups, and traditional methods such as stratification or matching are relatively blunt instruments that can only deal with a very small number of site characteristics. Sequential balancing of site characteristics can accommodate multiple features simultaneously and allows differential weighting of characteristics in arriving at a site assignment schema with minimal imbalance. We have applied long-established principles to develop a sequential balancing method for a SWD implementation trial.

## Abbreviations

BHIP-CCM: Behavioral Health Interdisciplinary Program-Collaborative Care Model
BIB: Balanced incomplete block
IMB: Imbalance score
SWD: Stepped wedge design
